# Long-Term Care Nurses’ Intent to Stay, Job Characteristics, and Organizational Justice : A Structural Equation Model

**DOI:** 10.1093/geroni/igaa057.279

**Published:** 2020-12-16

**Authors:** Lin Wang, Miao Miao, Xiaoping Yu

**Affiliations:** 1 Shanghai Jiao Tong University, Shanghai, China; 2 Ruijin Hospital, Shanghai, China

## Abstract

Background：Gratifying the elderly health-care demands and promoting high-quality of nursing services are based on the effort of long-term care(LTC) nurses. However, the high turnover rate of LTC nurses has become very serious in China. What remains unclear is whether the organizational justice and job characteristics affect the LTC nurses’ intention to stay. Objective: The aim was to investigate intention to stay among LTC nurses in relation to organizational justice and job characteristics. Method: A cross-sectional study was conducted with a convenience sample of 545 LTC nurses. Data collection was performed between July and November 2019. Data were analyzed using structural equation modeling. Results: Most of LTC nurses reported to stay in nursing or current work place, however they still had strong desire to leave if there were other job opportunities. Organizational justice and job characteristics were significant predictors of LTC nurses intent to stay. LTC nurses job characteristics partially mediates the relationship between organizational justice and intent to stay. Conclusion: This would suggest the importance of administrators/ managers understanding how to promote organizational justice, foster a justice climate and increase LTC nurses’ perceived job characteristics. The organizational justice culture programs should be develop as LTC nurses retention strategy.

